# Discovery of piperonal-converting oxidase involved in the metabolism of a botanical aromatic aldehyde

**DOI:** 10.1038/srep38021

**Published:** 2016-12-01

**Authors:** Shiori Doi, Yoshiteru Hashimoto, Chiaki Tomita, Takuto Kumano, Michihiko Kobayashi

**Affiliations:** 1 Institute of Applied Biochemistry, and Graduate School of Life and Environmental Sciences,The University of Tsukuba, 1-1-1 Tennodai, Tsukuba, Ibaraki 305-8572, Japan

## Abstract

Piperonal-catabolizing microorganisms were isolated from soil, the one (strain CT39-3) exhibiting the highest activity being identified as *Burkholderia* sp. The piperonal-converting enzyme involved in the initial step of piperonal metabolism was purified from strain CT39-3. Gene cloning of the enzyme and a homology search revealed that the enzyme belongs to the xanthine oxidase family, which comprises molybdoenzymes containing a molybdopterin cytosine dinucleotide cofactor. We found that the piperonal-converting enzyme acts on piperonal in the presence of O_2_, leading to formation of piperonylic acid and H_2_O_2_. The growth of strain CT39-3 was inhibited by higher concentrations of piperonal in the culture medium. Together with this finding, the broad substrate specificity of this enzyme for various aldehydes suggests that it would play an important role in the defense mechanism against antimicrobial compounds derived from plant species.

Generally, higher plant species secrete carbohydrates, amino acids, vitamins and other nutrients produced by themselves. Therefore, there are large numbers of microorganisms around plants, especially plant roots, in order to obtain nutrients. Some microorganisms in the plant rhizosphere can convert nutrients composed of carbon, nitrogen, etc. into useful compounds for plants, and others can degrade toxic compounds against plants into nontoxic ones, resulting in symbiosis between plants and microorganisms. On the other hand, plant pathogenic microorganisms are able to infect host plant cells or grow by use of compounds produced by plants. Although plants defend themselves immediately against microbial infections through a defense mechanism, resulting in the prevention of infection in most cases, some microorganisms have the abilitiy to avoid the plant defense responses. As a result, there are an enormous number of plant-pathogen interactions that remain to be elucidated.

Aromatic aldehydes are some of the components of essential oils, and are produced as secondary metabolites by aromatic plants. In the essential oils, which are volatile, natural, complex compounds characterized by a strong odour, there are numerous compounds for example, terpenes, terpenoides and aromatic compounds. It is commonly assumed that essential oils play an important role in the protection of plants as antibacterials, antivirals, antifungals and insecticides, and also against herbivores by reducing their appetite for such plants. They also may attract some insects, facilitatory dispersion of pollen and seeds, or repel undesirable ones^1^. However, little is known regarding the role of botanical volatile aromatic aldehydes in the essential oils derived from higher plants^1^,^2^, and findings regarding the biological metabolism of aromatic aldehyde compounds are particularly limited^3^. Here, we focused on a floral volatile secondary metabolite, piperonal, produced by the *Heliotropium* genus. Piperonal is known as a component of the essential oil of the heliotrope flower, and is an aldehyde frequently used in perfumes, cosmetics and flavoring agents, such as cinnamaldehyde and vanillin included in cinnamon spice and vanilla essence, respectively. However, the metabolic pathway of piperonal is unknown. In present article, we report the isolation of microorganisms with piperonal-converting ability from soil, the purification and characterization of a piperonal-converting enzyme, and the cloning of its gene. We also demonstrate that an O_2_-dependent enzyme is involved in piperonal metabolism.

## Results

### Isolation and identification of a piperonal-converting microorganism

After about one month from the start of the screening, using the acclimatization culture method described under “*Isolation of piperonal-converting bacteria”, w*e isolated 77 microorganisms with piperonal-converting ability from the soil. From among them, the microorganism (CT39-3) showing the highest activity was selected, as described under “*Assaying of piperonal-converting abilities of the isolated strains”.*

**Figure 1:**
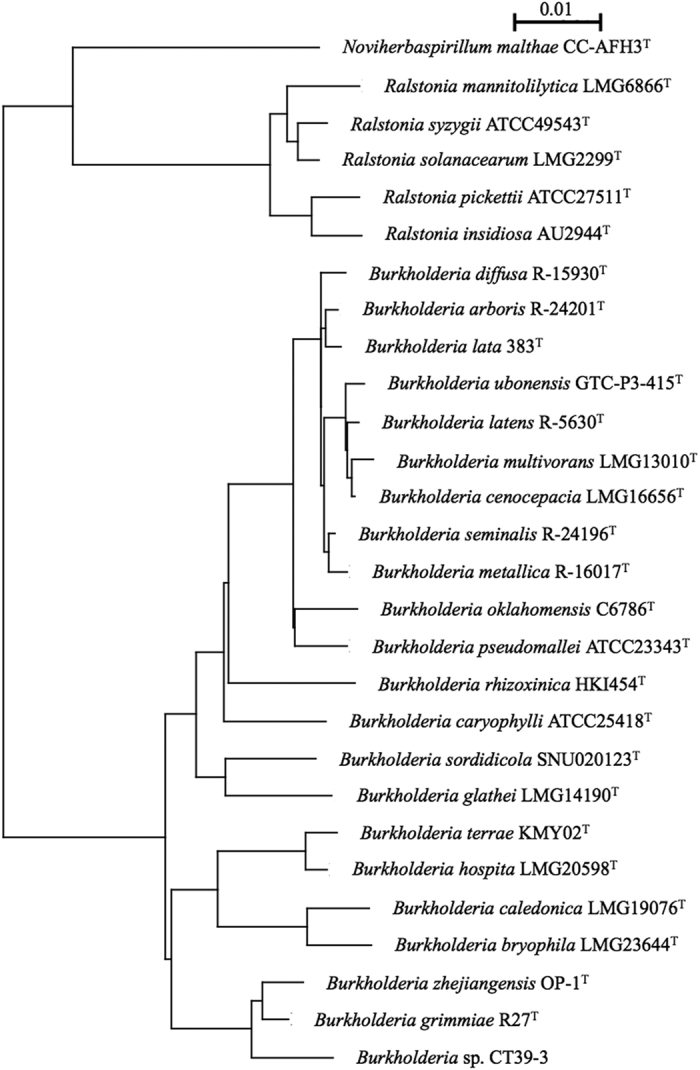
Phylogenetic tree of 16 S rRNA genes from *Burkholderia* sp. CT39-3 and its relatives. The scale bar represents 1% dissimilar sequences.

Morphologically, strain CT39-3 is a Gram-negative rod, nonendospore forming, and motile. Its physiological characteristics are as follows: nitrate reduction, positive; indole production, negative; acid production; glucose, negative; arginine dihydrolase, negative; urease, negative; esculine hydrolysis, negative; gelatin liquefaction, negative; β-galactosidase, negative; cytochrome oxidase, negative; starch hydrolysis, negative; lecithinase, negative; hipprate hydrolysis, negative; growth on sole carbon sources, positive with dextrose, l-arabinose, d-mannol, d-mannitol, l-rhamnose, l-malic acid, phenylacetic acid and *N*-acetylglucosamine, and negative with d-ribose, d-xylose, adipic acid, capric acid and gluconic acid. The 16 S rRNA gene sequence of the isolated microorganism was used as a query to search for homologous sequences in the Ribosomal Database Project (RDP)^4^. As a result, the nucleotide sequence of its 16 S rRNA gene was found to show 98.6% similarity to that of the closest type strain, *Burkholderia grimmiae* R27^T^, and 98.2% similarity to that of *Burkholderia zhejiangensis* OP-1^T^ 5. Phylogenetic analysis revealed that the strain is closely related to the genus *Burkholderia* (see Fig. 1). Based on these characteristics together with its 16 S rRNA sequence, strain CT39-3 was identified as *Burkholderia* sp.

### Identification of the reaction product

The product derived on piperonal conversion was analyzed by HPLC. First, the reaction was carried out for 30 min at 25 °C using an excess of the partially purified enzyme in standard mixture A described under “*Enzyme assays”*. In this case, only one peak with the consumption of piperonal was detected at 254 nm with the HPLC method. The retention time and UV-vis absorption spectrum of the reaction product were found to agree with those of authentic piperonylic acid (2.4 min). Thus, one of the reaction products was identified as piperonylic acid, but the other product remained unknown.

### Purification of the piperonal-converting enzyme

**Figure 2:**
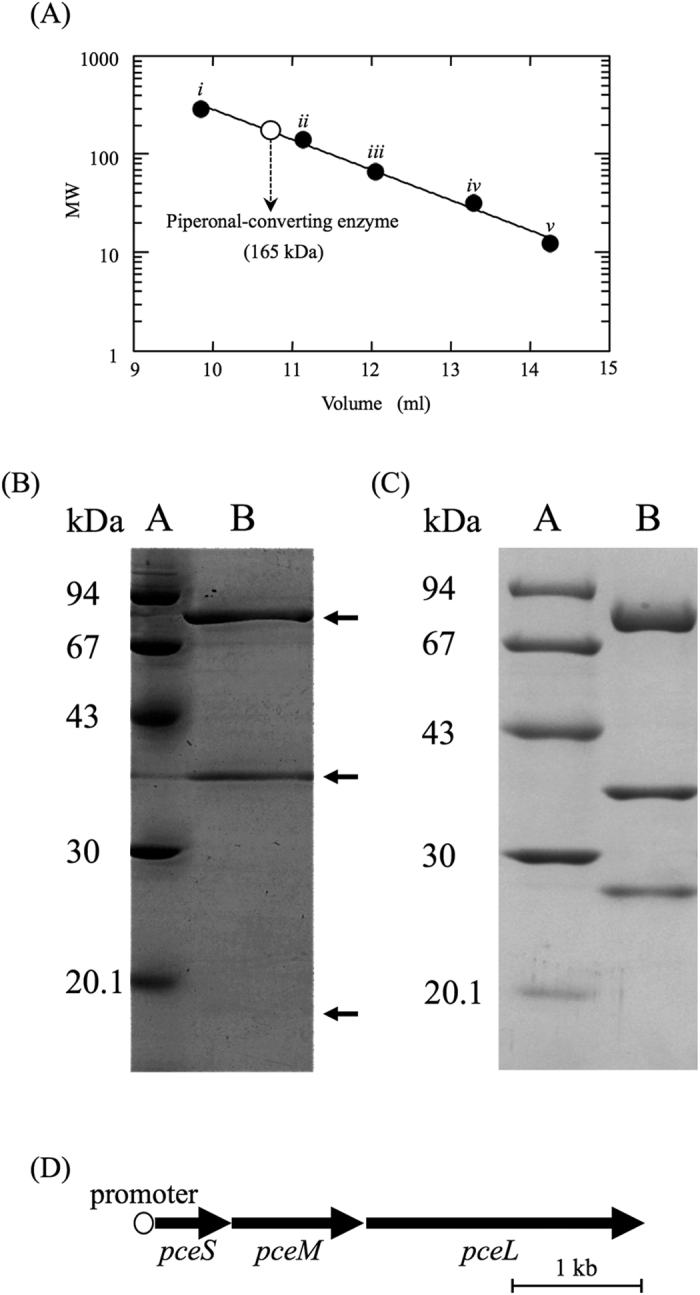
Purification of the piperonal-converting enzyme. (**A**) Molecular mass determination of the purified piperonal-converting enzyme. Marker proteins used for gel filtration: (*i*) glutamate dehydrogenase (yeast) (290 kDa); (*ii*) lactate dehydrogenase (pig heart) (142 kDa); (*iii*) enolase (yeast) (67 kDa); (*iv*) myokinase (yeast) (32 kDa); and (*v*) cytochrome c (horse heart) (12.4 kDa). The molecular mass determined is shown by an open circle. (**B**) SDS-PAGE of the purified piperonal-converting enzyme. Protein bands were detected by staining with Coomassie brilliant blue. *Lane A*, marker proteins. *Lane B*, the purified piperonal-converting enzyme. (**C**) SDS-PAGE of the purified recombinant piperonal-converting enzyme. Protein bands were detected by staining with Coomassie brilliant blue. *Lane A*, marker proteins. *Lane B*, the purified recombinant piperonal-converting enzyme. (**D**) The gene organization of the piperonal-converting enzyme.

From *Burkholderia* sp. CT39-3 cultured under optimal culture conditions we established (Table S1 and *SI Results*), the piperonal-converting enzyme was purified with a yield of 0.044%, through the purification steps described under “*Methods”*. The purified enzyme showed specific activity of 54.7 units/mg (Table 1). The purity of the enzyme was confirmed by elution of the protein as a single peak corresponding to 165 kDa on gel filtration chromatography (Fig. 2A and “*SI Methods*”). The purified enzyme gave three bands corresponding to molecular masses of 20 kDa, 40 kDa, and 80 kDa on SDS-PAGE (Fig. 2B). These findings indicate that the piperonal-converting enzyme consists of three subunits of these sizes.

### Cloning of the gene encoding the piperonal-converting enzyme

To identify the gene encoding the piperonal-converting enzyme, the N-terminal amino acid sequence of each of the 20 kDa, 40 kDa, and 80 kDa subunits of the purified enzyme was determined, as described under “*Methods”*. A local BLAST search was run on the draft genome database for strain CT39-3 constructed in-house, using the respective amino acid sequences. As a result, three ORFs (*piperonal-converting enzyme*) coding for the 20 kDa, 40 kDa, and 80 kDa subunits were found, which were located adjacently in the same transcription direction (Fig. 2D). The gene (named *pceS*) encoding the 20 kDa subunit consists of 561 nucleotides and codes for a protein of 186 amino acids with a calculated molecular mass of 19,576 Da. The gene (named *pceM*) encoding the 40 kDa subunit consists of 1,008 nucleotides and codes for a protein of 335 amino acids with a calculated molecular mass of 35,948 Da. The gene (named *pceL*) encoding the 80 kDa subunit consists of 2,226 nucleotides and codes for a protein of 741 amino acids with a calculated molecular mass of 78,956 Da. Each of the calculated molecular masses was consistent with that of each subunit of the purified enzyme determined on SDS-PAGE. The predicted promoter sequences, -35 (TCGCCG) and -10 (CTGCACAAT), in the region upstream of the ATG initiation codon of *pceS* indicated that three genes (*pceSML*) may be transcribed as one operon from that promoter.

A search with the BLAST program revealed that the deduced amino acid sequence of the (PceSML): the deduced amino acid sequence of PceS exhibits 71% similarity with 2Fe-2S iron-sulfur cluster binding domain-containig protein (fer2 superfamily and Fer2–2 superfamily) and CoxS (S subunit of carbon monoxide dehydrogenase); the deduced amino acid sequence of PceM exhibits 77% similarity with molybdopterin dehydrogenase FAD-binding (FAD-binding-4 superfamily) and CoxM (M subunit of carbon monoxide dehydrogenase); and the deduced amino acid sequence of PceL exhibits 77% similarity with those of aldehyde oxidase, xanthine dehydrogenase containing molybdopterin and CoxL (L subunit of carbon monoxide dehydrogenase)^6^.

### Expression and purification of the recombinant piperonal-converting enzyme

In order to overproduce the piperonal-converting enzyme, an expression plasmid (pBBR-His-*pceSML*) was constructed and introduced into *Burkholderia* sp. CT39-3 described under *“SI Methods”*. Cell-free extracts prepared from the strain CT39-3 transformant carrying pBBR-His-*pceSML*, which was cultured at 28 °C, rapidly converted piperonal (as a substrate) into piperonylic acid. We analyzed cell-free extracts by SDS-PAGE, and detected remarkable protein bands at the 20 kDa, 40 kDa, and 80 kDa positions corresponding to three subunits of the enzyme purified from *Burkholderia* sp. CT39-3, respectively. On the other hand, these three remarkable bands were not detected for cell-free extracts prepared from strain CT39-3 carrying no expression plasmid. Therefore, overproduction of the piperonal-converting enzyme in the active form was attained.

The piperonal-converting enzyme produced in the recombinant cells was purified to homogeneity through the HisTrap HP column chromatography described under “*SI Methods”*. The specific activity of the purified enzyme was found to be 62.2 units/mg. The purified enzyme gave three bands on SDS-PAGE (Fig. 2C), this band pattern being almost the same as that of the enzyme purified from strain CT39-3 except for the smallest band representing the N-terminal His·tag fusion with PceS. These data indicated that the piperonal-converting enzyme purified from *Burkholderia* sp. CT39-3 carrying pBBR-His-*pceSML* is identical to that from strain CT39-3. Since the purified enzyme could not be obtained in a large amount from *Burkholderia* sp. CT39-3 and detailed analyses required a large amount of the enzyme, we used the recombinant piperonal-converting enzyme in the following experiments.

### Cofactor analysis of the piperonal-converting enzyme

Purified PceSML was brownish yellow in solution and exhibited homology with molybdo-flavoenzymes, e.g., xanthine oxidase. Thus, the existence of molybdopterin (MPT), flavin and [2Fe-2S] cluster as cofactors in PceSML was expected.

**Figure 3:**
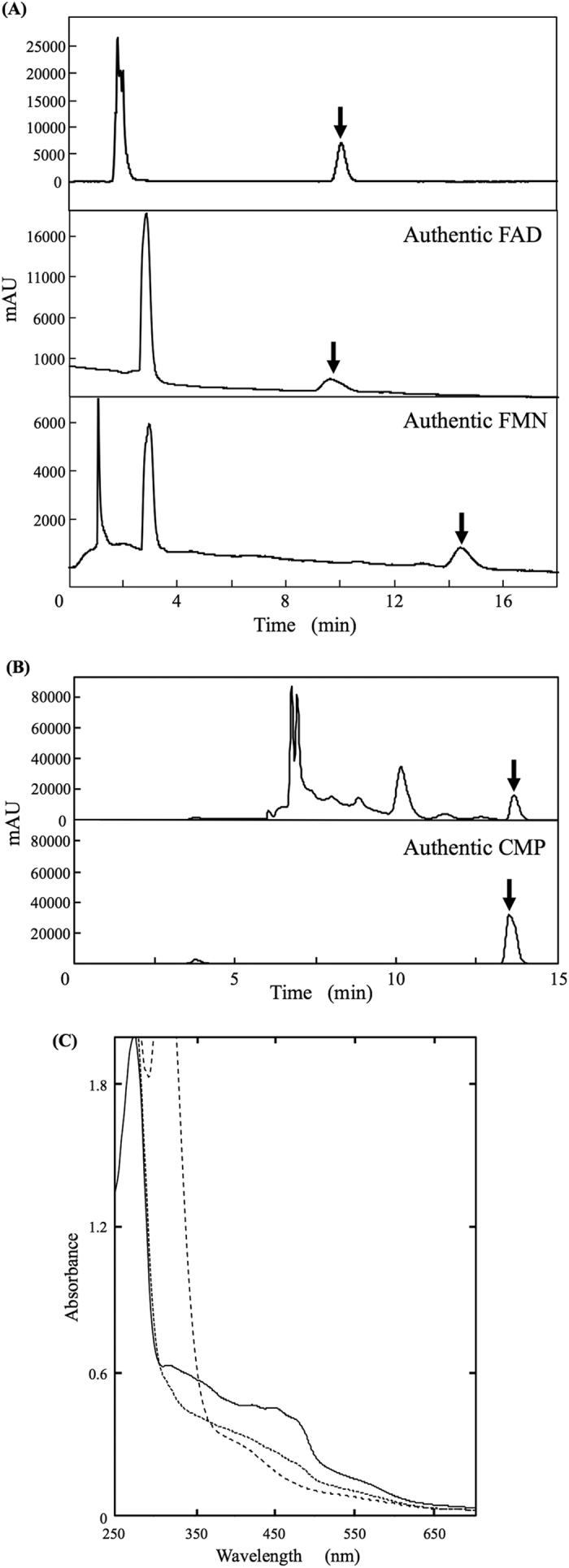
Cofactor analyses of the purified piperonal-converting enzyme. (**A**) HPLC elution profiles of the reaction mixture containing the purified enzyme and HClO_4_ (top), authentic FAD (middle), and authentic FMN (bottom). (**B**) HPLC elution profiles of the reaction mixture containing the purified enzyme and H_2_SO_4_ (top), and authentic CMP (bottom). (**C**) Absorption spectra of the piperonal-converting enzyme. The purified enzyme (solid line); the enzyme incubated with 2 mM benzaldehyde (dotted line); and the enzyme reduced with 10 mM sodium dithionite (dashed line). The concentration of enzyme protein was 2.0 mg/ml (250–700 nm) in 100 mM KPB (pH 7.5) and 1 mM EDTA. The same buffer was examined as a control. Absorption spectra were measured at room temperature.

First, in order to clarify whether the flavin cofactor is flavin-adenine-dinucleotide (FAD) or flavin-mononucleotide (FMN), an equal amount of 10% HClO_4_ was added to the enzyme to release the flavin cofactor from PceSML and then the supernatant containing flavin was analyzed by HPLC. As a result, FAD was identified as the flavin cofactor (Fig. 3A), and its content was calculated to be 0.88 mol/mol of PceSML.

In order to identify the nucleotide bound to MPT in the enzyme by HPLC, purified PceSML was incubated for 15 min in the presence of sulfuric acid, which can release the nucleotide of the molybdopterin dinucleotide cofactor, as described under *“SI Methods”*^7^. As a result, cytidine monophosphate (CMP) was detected (Fig. 3B), the content of which was 0.61 mol/mol of PceSML. Quantitative analysis of metals (Mo and Fe) in the purified PceSML with an ICP emission spectroscope (ICPS-8100) revealed that PceSML contained Mo (0.55 mol/mol of PceSML). The content of CMP included in MPT corresponded well to that of Mo in PceSML. Furthermore, the enzyme contained Fe (5.2 mol/mol of PceSML), suggesting that there was enough Fe to comprise the two [2Fe-2S] clusters per enzyme. On the basis of these results, the piperonal-converting enzyme would contain FAD, molybdopterin cytosine dinucleotide cofactor (MCD), and [2Fe-2S] cluster in a ratio of 1:1:2 per PceSML.

### UV-visible spectral analysis of the piperonal-converting enzyme

The purified enzyme exhibited an absorption spectrum with maxima at 320, 360, 420, 450, 470, and 560 nm (Fig. 3C), this being similar to the absorption spectra of other molybdo-flavoenzymes^7^,^8^. These peaks were suggested to be derived from the cofactors. The piperonal-converting enzyme gave cofactor absorption peaks at the following wavelengths: flavin absorption maxima at 386 nm and around 450 nm, and [2Fe-2S] center absorption around 325, 420, 465, and 550 nm^9^. The spectrum of PceSML on the addition of sodium dithionite (Fig. 3C) showed that the enzyme was completely reduced. In order to obtain a spectrum with the addition of substrate, benzaldehyde was used instead of piperonal, because piperonal exhibited its own absorption peaks at around 300 nm that prevented the measurement of a UV-visible spectrum. The resultant spectrum was different from those in the absence and presence of sodium dithionite, indicating that PceSML was partially reduced on the addition of a substrate. During the reduction of PceSML with either benzaldehyde or sodium dithionite, a peculiar spectrum derived from the formed flavin semiquinone was not observed.

### Identification of another substrate and another reaction product, and stoichiometry

**Figure 4:**
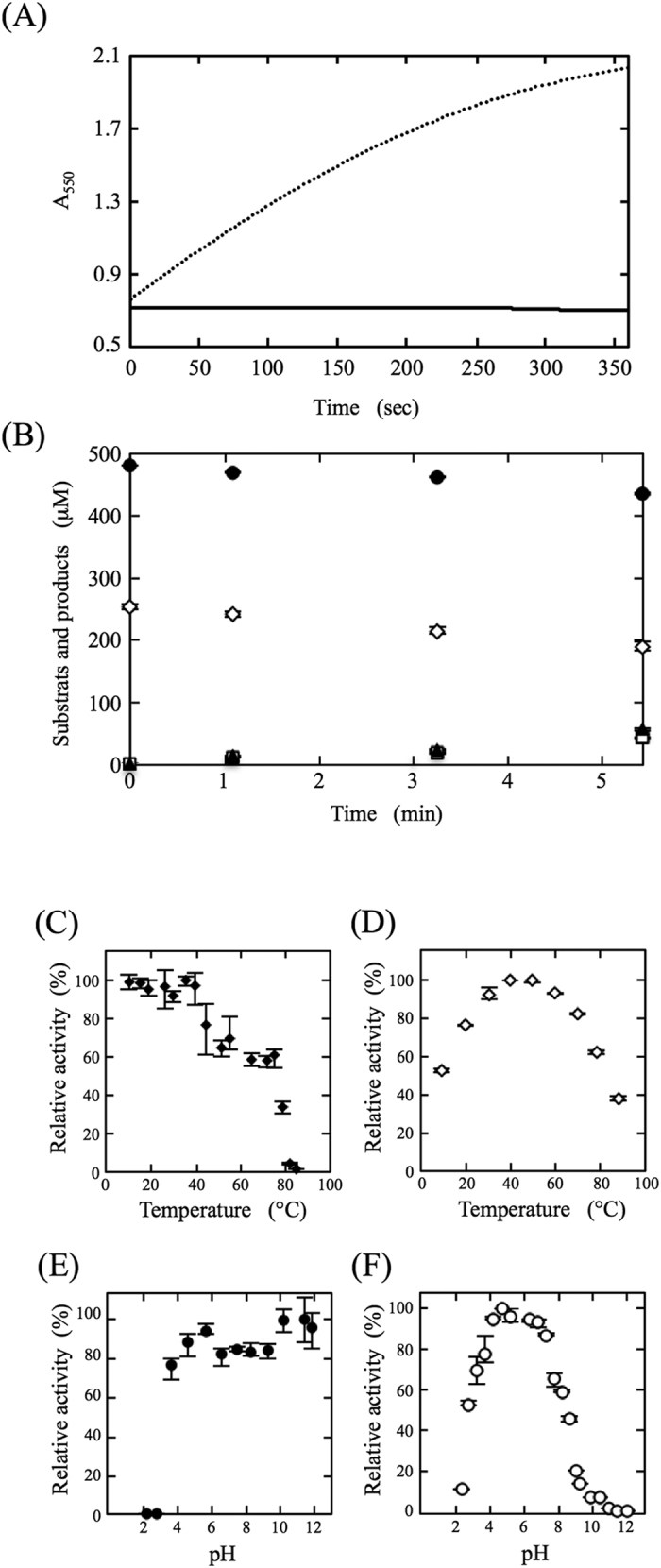
Characterization of the piperonal-converting enzyme. (**A**) Assay for the generation of superoxide production during enzyme reactions. 0.03 units PceSML and 0.5 mM piperonal (solid line); 0.03 units xanthine oxidase from butter milk and 0.5 mM xanthine (dotted line). (**B**) Stoichiometry analysis of the piperonal-converting reaction. ●, piperonal; ◊, O_2_; ▲, piperonylic acid; □, H_2_O_2_. The procedures for the analysis of reactants and products are described under *“Methods”*. (**C**) Reactions were carried out at various temperatures. (**D**) The enzyme was preincubated at various temperatures for 30 min in 0.1 M KPB (pH 7.5), an aliquot of each enzyme solution was taken, and then the enzyme activity was assayed under the “standard assay A” conditions. (**E**) Reactions were carried out in Britton-Robinson buffer (0.1 M) at different pH values. (**F**) The enzyme was preincubated at various pH values at 25 °C for 30 min in Britton-Robinson buffer at a concentration of 0.1 M, an aliquot of each enzyme solution was taken, and then the enzyme activity was assayed under the “standard assay A” conditions. The relative activity is expressed as the percentage of the maximum activity attained under the experimental conditions used. The values represent the means ± SD at least five independent experiments.

Based on the identification of one of the reaction products as piperonylic acid and the results of the homology search with the piperonal-converting enzyme, another substrate and other reaction products were suggested to be O_2_, H_2_O_2_, and superoxide (O_2_^•−^), respectively. First, consumption of O_2_ was analyzed with an oxygen electrode using the “standard assay B” conditions. A significant decrease of O_2_ in the reaction mixture was demonstrated, indicating that another substrate was O_2_. Next, production of O_2_^•−^ was analyzed by means of the cytochrome *c* reduction assay described under *“SI Methods”*^10^,^11^. As a result, no increase of O_2_^•−^ in the reaction mixture was detected. On the other hand, production of O_2_^•−^ was detected in the reaction mixture containing xanthine oxidase and xanthine instead of the piperonal-converting enzyme and piperonal (Fig. 4A). These findings demonstrated that O_2_^•−^ was not the reaction product of PceSML. Third, production of H_2_O_2_ was analyzed by HPLC, an increase in H_2_O_2_ produced in the reaction mixture being detected. Thus, another reaction product was identified as H_2_O_2_.

Using the purified enzyme, the stoichiometry of consumption of the substrates and formation of the reaction products during the enzyme reaction was examined in a reaction mixture consisting of 100 mM KPB (pH 7.5), 0.5 mM piperonal and 0.01 mg/ml enzyme. The reaction was carried out at 25 °C. The amounts of residual piperonal, residual O_2_, formed piperonylic acid and formed H_2_O_2_ in the reaction mixture were determined to be 451, 231, 12 and 9 μmol, respectively, when the initial amounts of piperonal and O_2_ as the substrates were 462 and 243 μmol, respectively (Fig. 4B). The results demonstrated that piperonylic acid and H_2_O_2_ were formed stoichiometrically with the consumption of piperonal and O_2_ during the enzyme reaction, and that O_2_ acted as a terminal electron acceptor.

### Effects of various electron acceptors on the enzyme activity

We tested other electron acceptors, i.e., 2,6-dichlorophenol-indophenol, benzoquinone, cytochrome *c*, NAD^+^, and ferredoxin, under anaerobic conditions, namely, in the absence of O_2_. No enzyme activity was detected when cytochrome *c*, NAD^+^, or ferredoxin was used as the electron acceptor (Table 2). On the other hand, 2,6-dichlorophenol-indophenol and benzoquinone were able to act as electron acceptors for the enzyme. However, the enzyme activity with O_2_ as the electron acceptor was the highest, and the relative activity with 2,6-dichlorophenol-indophenol and benzoquinone was 13% and 26%, respectively (Table 2).

### Substrate specificity

PceSML could convert almost all aldehyde compounds regardless of whether they were aromatic or aliphatic ones (Table 3). On the other hand, formaldehyde and xanthine were inert. Among all the tested aldehydes, piperonal showed the highest catalytic efficiency, i.e., a *k*_*cat*_/*K*_*m*_ value of 7700 with the lowest *K*_*m*_ value of 0.019 ± 0.002 mM. Cinnamoaldehydes and *m*-hydoroxybenzaldehyde also showed higher *k*_*cat*_/*K*_*m*_ values of 6200 and 5600, respectively. These results indicate that the piperonal-converting enzyme is an aldehyde oxidase with preference for aromatic or hydrophobic aldehydes.

### Effects of temperature and pH on the stability and activity of the enzyme

The effects of temperature and pH on the enzyme stability and activity were examined. The stability of the enzyme was investigated at various temperatures. After the enzyme had been preincubated for 30 min in 10 mM KPB (pH 7.5), an aliquot of the enzyme solution was taken and then the enzyme activity was assayed under the “standard assay A” conditions. The enzyme was most stable at from 10 to 62 °C (Fig. 4C). The optimal reaction temperature appeared to be 40 °C (Fig. 4D). At higher than 50 °C, the specific activity gradually decreased. As the reaction temperature became higher, the concentration of O_2_ dissolved in the reaction mixture decreased under the assay conditions used. It was suggested that the concentration of O_2_ dissolved in the reaction mixture might have affected the specific activity, which became lower depending on the reaction temperature at higher than 50 °C.

After the enzyme had been incubated at 25 °C for 30 min in the 0.1 M Britton-Robinson buffer (pH 2.2–11.9)^12^, an aliquot of the enzyme solution was taken and then the enzyme activity was assayed under the “standard assay A” conditions. The enzyme was most stable in the pH range of 4.6 to 11.9, with 80% of the initial activity being retained even at pH 11.9 (Fig. 4E). The enzyme exhibited maximum activity at pH 4.8 (Fig. 4F).

### Growth of Burkholderia sp. CT39-3 in the minimal media containing different carbon sources

**Figure 5:**
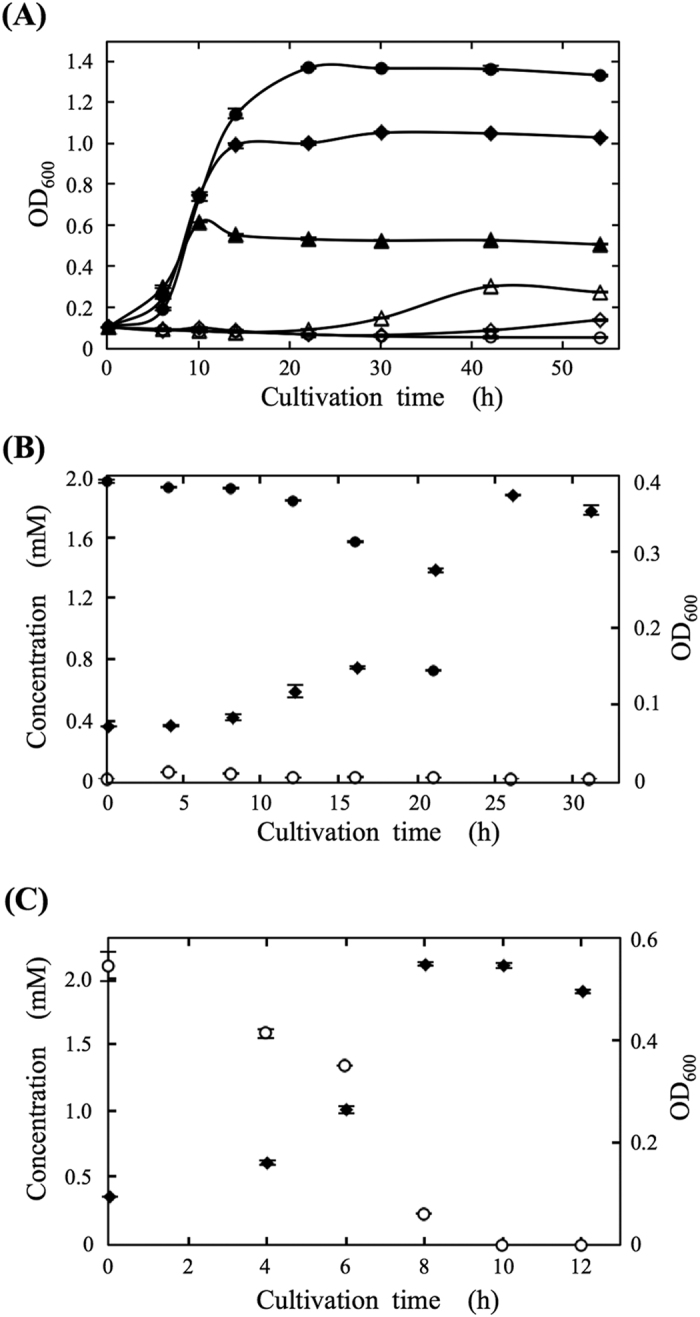
Growth of *Burkholderia* sp. CT39-3 in the minimal media containing different carbon sources. (**A**) Cell growth of *Burkholderia* sp. CT39-3 cultured with 0.03% piperonal (△), 0.07% piperonal (◊), 0.10% piperonal (○), 0.03% glucose (▲), 0.07% glucose (♦), or 0.10% glucose (●). (**B**) Cell growth of *Burkholderia* sp. CT39-3 (♦), concentration of piperonal in a medium (●), and concentration of piperonylic acid in a medium (○). (**C**) Cell growth of *Burkholderia* sp. CT39-3 (♦), concentration of piperonylic acid in a medium (○). Aliquots of the culture were removed at three independent cultures at each time point. Each concentration of both piperonal and pipernylic acid was measured by HPLC and each growth was measured by determining the average optical density (OD_600_). The values represent the means ± SD at least three independent experiments.

To determine the function of the piperonal-converting enzyme *in vivo, Burkholderia* sp. CT39-3 was grown in several minimal culture media. *Burkholderia* sp. CT39-3 exhibited full growth in the medium containing glucose as the sole carbon source (Fig. 5A), piperonal-converting enzyme activity being observed. On the other hand, even on replacement of glucose with 0.03% (w/v) piperonal, *Burkholderia* sp. CT39-3 grew (Fig. 5A). During the lag growth phase, the amount of piperonal decreased stoichiometrically with an increase in the amount of piperonylic acid in the culture medium. Then, the amounts of piperonal and piperonylic acid in the medium decreased with the growth of strain CT39-3. Finally, both piperonal and pipernonylic acid in the medium were completely consumed in the stationary phase (Fig. 5B). As shown in Fig. 5A, however, the bacterial growth became substantially delayed as the concentration of piperonal increased (0.07%) and was completely inhibited on the addition of 0.10% piperonal. The piperonal-converting enzyme activity was also detected in the cells grown in the medium containing 0.03% or 0.07% piperonal.

Moreover, strain CT39-3 was grown in the medium containing piperonylic acid as a sole carbon source in the same way to as above-mentioned. Piperonylic acid in the medium decreased during the logarithmic growth phase and was completely consumed in the stationary phase (Fig. 5C).

## Discussion

For a long time, human beings have ingested primary metabolites produced by plants as nutrients, and have also utilized various secondary ones as potential drugs. It has been reported that there are almost 250 thousand plants including higher ones on earth. Therefore, there is an enormously diverse range of specific secondary metabolites depending on the species or group. Degradation of various kinds of secondary metabolites produced by higher plants has been reported. For example, caffeine, a kind of alkaloid present in coffee, is degraded by several enzymes derived from microorganisms in nature^13^,^14^,^15^. Polyphenol curcumin, a yellow pigment, derived from the rhizomes of a plant (*Curcuma longa* Linn), is metabolized by gut bacteria^16^. Sesamin found in sesame undergoes microbial degradation by soil actinomycete^17^. Although a lot of microorganisms showing degradation ability as to plant secondary metabolites have been isolated, studies on enzymes involved in the degradation pathways have been limited.

Piperonal is a naturally occurring aromatic aldehyde, a secondary metabolite produced by higher plants, and well-known as a volatile compound frequently used in perfumes, cosmetics and flavoring agents^18^. To the best of our knowledge, there has been neither isolation of microorganisms nor elucidation of the metabolic pathway involved in piperonal metabolism. Furthermore, the enzymes, and their genes, involved in the degradation of piperonal remain unclear.

In this work, we isolated many piperonal-catabolizing microorganisms and chose one, CT39-3, which was identified as *Burkholderia* sp., from soil. Although this strain showed the highest activity as to piperonal conversion among the isolated microorganisms, the amount of cells cultured in the screening medium was extremely small, and the piperonal-converting activity was low in the cells. Thus, we established the optimum culture conditions and purified the enzyme from a large amount of cells (108 L) cultured under these conditions. The purified enzyme gave three bands corresponding to molecular masses of 20 kDa, 40 kDa, and 80 kDa on SDS-PAGE (Fig. 2B). This piperonal-converting enzyme has been found to act on piperonal in the presence of O_2_, leading to formation of piperonylic acid and H_2_O_2_. The formation of no other compounds was observed. Piperonylic acid and H_2_O_2_ were formed with the consumption of piperonal and O_2_ in a 1:1:1:1 stoichiometry. We, for the first time, clarified the initial step of piperonal metabolism and identified the enzyme involved in the metabolism.

In general, oxidoreductases that convert an aldehyde to the corresponding acid utilize an electron acceptor for the reaction. For instance, aldehyde dehydrogenases (EC 1.2.1.3, 1.2.1.4, and 1.2.1.5) use NAD and/or NADP as a coenzyme. *E. coli* periplasmic aldehyde oxidoreductase might use ferredoxin as a physiological electron acceptor^7^. Previously, an aldehyde oxidoreductase from a sulfate-reducing bacterium of genus *Desulfovivrio* using 2,6-dichlorophenol-indophenol as an electron acceptor was isolated and characterized^19^. In the present study, we tested various electron acceptors in order to identify the physiological one for PceSML. Among the natural compounds, only molecular oxygen, O_2_, acted as an electron acceptor for the enzyme. Although benzoquinone and 2,6-dichlorophenol-indophenol were able to act as electron acceptors, both are artificial ones and their activities were much lower than the activity with O_2_. These findings indicate that the physiological electron acceptor for PceSML is O_2_, and that the piperonal-converting enzyme is an oxidase, but not a dehydrogenase.

Aldehyde oxidoreductase is a member of the xanthine oxidoreductase family, which consists of xanthine oxidoreductase, ferredoxin oxidoreductase, carbon monoxide dehydrogenase, quinoline 2-oxidoreductase, and so on. These enzymes are very important due to their primordial roles in bacterial cells. The overall amino acid sequence similarity between aldehyde oxidoreductases and xanthine oxidoreductases is approximately 50%, clearly indicating that both types of enzyme originated from a common ancestral precursor^20^. The biochemical and physiological functions of xanthine oxidoreductases were revealed to be in purine catabolism: conversion of hypoxanthine to xanthine and subsequent conversion of xanthine to uric acid. On the other hand, those of aldehyde oxidoreductases, which exist in the organs of man, archaea, bacteria and so on, have remained largely unclear^20^. Also, the physiological roles of aldehyde oxidases belonging to the aldehyde oxidoreductase family are not yet understood in detail, but they might have some significant roles in bacteria. Based upon the substrate specificity results, the piperonal-converting enzyme was found not to be a xanthine oxidase involved in purine metabolism. In order to determine the physiological function of PceSML, the growth of strain CT39-3 was examined under various culture conditions. In the medium containing 0.03% piperonal as the sole carbon source, piperonal decreased and piperonylic acid stoichiometrically increased, even during the lag growth phase of strain CT39-3 (Fig. 5B). Considering that piperonal would have antimicrobial activity against strain CT39-3 because of the inhibition of the bacterial growth on the addition of a higher concentration of piperonal (Fig. 5A), strain CT39-3 might detoxify piperonal into less toxic piperonylic acid. As strain CT39-3 grew, piperonal further decreased and was eventually consumed completely, indicating that strain CT39-3 metabolizes both piperonal and piperonylic acid as energy sources. In the medium containing 0.03% piperonylic acid as a carbon source, piperonylic acid was utilized. This observation further proves that the strain CT39-3 can metabolize piperonylic acid as an energy source. These results indicate that *Burkholderia* sp. CT39-3 is able to assimilate piperonal by means of the piperonal-converting enzyme via piperonylic acid. Therefore, the physiological role of the piperonal-converting enzyme would be the metabolism of various aldehyde compounds and/or their detoxification. In particular, the apparent *K*_*m*_ value for piperonal is extremely low and its *k*_*cat*_/*K*_*m*_ value is the highest, these values being reasonable if the purified enzyme is considered to be involved in the utilization and/or detoxification of piperonal *in vivo*. Some aromatic aldehydes, for example, vanillin and cinnamaldehyde, have been shown to have antimicrobial properties^7^. Thus, it has been reported that aromatic aldehydes contribute to the defense mechanisms against bacterial pathogens in plants due to their antimicrobial abilities^1^,^2^. Some *Burkholderia* species have been reported to be phytopathogenic bacteria, and to exist on the surface of plants or in soil^21^,^22^. Considering that strain CT39-3 was identified as *Burkholderia* sp. and its growth was inhibited by piperonal. Growth of some bacteria was inhibited by piperonal at low concentration, indicating that piperonal acts as an antimicrobial compound (*SI Results*). It is suggested that the piperonal-converting enzyme would play an important role in the defense mechanism against the antimicrobial compounds derived from plant species, opening new avenues for further investigation of the relationship between plants and microorganisms.

Members of the molybdoenzyme family in bacteria need post-translational maturation processes, such as Fe-S cluster biosynthesis, FAD-binding, and incorporation of molybdopterin^23^. For a final holoenzyme, molecular chaperones are involved^20^. In bacterial genomes, it has been reported that structural genes encoding members of the aldehyde oxidoreductase family are clustered with those of the corresponding molecular chaperones. Based on comparison of the piperonal-converting enzyme with other aldehyde oxidoreductases, and on the results of cofactor and spectroscopic analyses of the purified enzyme, the piperonal-converting enzyme is suggested to contain two [2Fe-2S] clusters, FAD and molybdopterin cytosine dinucleotide. As for other aldehyde oxidoreductases, post-translational maturation of the piperonal-converting enzyme seems to be suggested. In strain CT39-3, the structural genes *pceS, pceM* and *pceL* are clustered, however, no genes are located between the promoter and *pceSML*, and another promoter for other gene(s) is located just downstream of *pceSML*. This gene organization indicates that the gene coding for the molecular chaperon for PceSML does not exist in either an upstream or downstream region. A search of the draft genome of strain CT39-3 revealed that some ORFs homologous to the *paoD* gene exist in a quite different locus of *pceSML*. One of them could be involved in the post-translational maturation of PceSML in the same manner as for other aldehyde oxidoreductases. Because these ORFs are not clustered with *pceSML*, we constructed an expression plasmid harboring only *the pceSML* genes and used strain CT39-3 as a host in order to overproduce the piperonal-converting enzyme.

Analysis of the substrate specificity of PceSML showed a broad substrate specificity for various aldehydes (Table 3). The *V*_*max*_ values of PceSML shown in Table 3 are high, therefore, almost all aldehydes are rapidly converted to the corresponding carboxylic acids without the addition of any coenzyme (e.g., NAD) to the reaction mixture. It is obvious that an O_2_-dependent piperonal-converting enzyme is significantly advantageous from an economical standpoint to produce carboxylic acids from aldehydes, rather than NAD(P)-dependent aldehyde oxidoreductases, because it does not require any expensive cofactors^24^. The purification of the piperonal-converting enzyme from the native strain CT39-3 was very difficult, because the yield of the enzyme was extremely low. On the other hand, the expression of the recombinant piperonal-converting enzyme is very high. Moreover, a large amount of the recombinant piperonal-converting enzyme can be obtained because a recombinant enzyme is easy to purify using an N-terminal His·tag fusion to PceS. Therefore, it is possible to produce carboxylic acids effectively in the reaction mixture using the resting cells or the recombinant piperonal-converting enzyme. The piperonal-converting enzyme exhibits great potential as to the bioconversion of various carboxylic acids from the corresponding aldehyde compounds in the future.

## Methods

Detailed descriptions of the materials, culture conditions for *Burkholderia* sp. CT39-3, molecular mass determination, plasmids, strains, and medium, DNA manipulations, preparation of *Burkholderia* sp. CT39-3 competent cells for electroporation, analytical methods, expression and purification of the recombinant piperonal-converting enzyme, genome sequencing of strain CT39-3, identification of the enzyme flavin cofactor, analysis of cytidine monophosphate cofactor, metal analysis, detection of O_2_^•−^ formation using the cytochrome *c* assay, substrate specificity and determination of minimum inhibitory concentration are given in “*SI Methods*”.

### Isolation of piperonal-converting bacteria

Piperonal-converting bacteria were isolated from soil samples as follows. A spoonful of a soil sample was added to a test tube containing 10 ml of a screening medium (pH 7.5) consisting of 0.1% (w/v) (NH_4_)_2_SO_4_, 0.05% (w/v) K_2_HPO_4_, 0.05 g KH_2_PO_4_, 0.2 g NaCl, 0.05% (w/v) MgSO_4_·7H_2_O, 0.001% (w/v) FeSO_4_·7H_2_O, and 0.01% (w/v) piperonal (final concentration). Then, 100 μl of the first subculture was inoculated into a test tube containing 10 ml of the screening medium containing 0.02% (w/v) piperonal (final concentration). The second subculture was inoculated into a test tube containing 10 ml of the screening medium containing 0.03% (w/v) piperonal (final concentration) in the same way as for the second subculture. All subcultures were carried out at 28 °C for 1 week with reciprocal shaking in a test tube containing 10 ml of the screening medium with stepwise increases in the piperonal concentration. After one month’s further cultivation, microorganisms were spread on agar plates and isolated.

### Assaying of piperonal-converting abilities of the isolated strains

Each of the isolated strains was inoculated into a test tube containing 10 ml of medium and then incubated at 28 °C for 1 week with reciprocal shaking. Then, the cells were harvested by centrifugation, washed twice with 10 mM potassium phosphate buffer (KPB) (pH 7.5), and suspended in 10 mM KPB (pH 7.5).

The piperonal-converting abilities of the isolated strains were assayed by means of the resting cell reaction. The reaction mixture (1 ml) was composed of 10 μmol of KPB (pH 7.5), 2 μmol of piperonal, and an appropriate amount of cell suspension. The reaction was carried out at 25 °C for 1 h, and stopped by adding an equal volume of acetonitrile to the reaction mixture and then rapidly removing the cells by centrifugation at 0–4 °C. The residual amount of piperonal in the reaction mixture was determined by HPLC with a Shimadzu LC-10A system (Kyoto, Japan) equipped with a Cosmosil 5C_18_-AR-II column (reversed-phase, 4.6 × 150 mm; Nacalai Tesque) and the diode array detector (SPD-M10Avp) of the original system. The following solvent system was used: acetonitrile/formic acid/H_2_O, 50:0.1:49.9 (v/v), at the flow rate 1.0 ml/min and 40 °C. The absorbance was measured at 254 nm. One unit of the piperonal-converting enzyme activity was defined as the amount of the enzyme that catalyzed the conversion of 1 μmol piperonal/min under the above conditions.

### Purification of a piperonal-converting enzyme

All purification procedures were performed at 0–4 °C. KPB (pH 7.5) was used throughout the purification. Centrifugation was carried out for 30 min at 13,000 × *g*.

#### Step 1. Preparation of a Cell-free Extract

Washed cells from 108 liters of culture were resuspended in 78 ml of 100 mM buffer and then disrupted by sonication at 200 W for 30 min with an Insonator model 201 M (Kubota, Tokyo, Japan). The cell debris was removed by centrifugation.

#### Step 2. Ammonium Sulfate Fractionation

The resulting supernatant solution was fractionated with ammonium sulfate (40–60% saturation), followed by dialysis against 10 mM buffer.

#### Step 3. TOYOPEARL Butyl-650 M Column Chromatography

The enzyme solution from step 2 was brought to 3 M KCl, and then placed on a TOYOPEARL Butyl-650 M column (2.6 × 22 cm; Tosoh Co., Ltd.) equilibrated with 10 mM buffer containing 3 M KCl. The enzyme was eluted by lowering the concentration of KCl (3 to 1.2 M saturation) in 240 ml of the same buffer. The active fractions were pooled and then dialyzed against 10 mM buffer.

#### Step 4. Ceramic Hydroxyapatite Type I Column Chromatography

The enzyme solution from step 3 was applied to a Ceramic Hydroxyapatite Type I column (5 ml; Bio-Rad Laboratories) equilibrated with 10 mM buffer. Protein was eluted from the column with 40 ml of the same buffer, the concentration of KPB being increased linearly from 10 mM to 300 mM. The active fractions were collected and then dialyzed against 10 mM buffer.

#### Step 5. Resource Q Column Chromatography

The enzyme solution from step 4 was applied to a Resource Q column (6 ml; GE Healthcare UK Ltd.) equilibrated with 10 mM buffer. Protein was eluted from the column with 35 ml of the same buffer, the concentration of KCl being increased linearly from 0 to 0.6 M. The active fractions were pooled and then dialyzed against 10 mM buffer.

#### Step 6. TSKgel BioAssist Q Column Chromatography

The enzyme solution from step 5 was applied to a Bioassist Q column (1 ml; Tosoh Co., Ltd.) equilibrated with 10 mM buffer. Protein was eluted from the column with 60 ml of the same buffer, the concentration of KCl being increased linearly from 0 to 0.5 M. The active fractions were pooled and then dialyzed against 10 mM buffer.

### Enzyme assays

Piperonal-converting activity was measured by means of the following two assay systems. All of the reactions were performed under linear conditions as to protein and time.

The standard assay A mixture comprised 100 mM KPB (pH 7.5), 2 mM piperonal, and an appropriate amount of enzyme, in a total volume of 200 μl. The reaction was started by the addition of piperonal and carried out at 25 °C. The reaction was stopped by adding 200 μl of 99% (v/v) acetonitrile and 1% (v/v) formic acid to the reaction mixture, and a supernatant was obtained by centrifugation (15,000 × *g*, 3 min). The amount of piperonylic acid formed was determined by HPLC, which was performed with the same system as used for the measurement of piperonylic acid under “*Assaying of piperonal-converting abilities of the isolated strains*”. This assay was used to routinely measure piperonal-converting activity, unless otherwise noted.

In the case of standard assay B, enzyme activity was assayed by measuring the decrease in O_2_ with an oxygen electrode (Hansatech Instruments Ltd., Norfolk, UK), which monitors the O_2_ concentration. The reaction mixture was composed of 2 mM aldehyde, 100 mM KPB (pH 7.5), and an appropriate amount of enzyme (10 μl), in a final volume of 1 ml, unless otherwise noted. The reaction was initiated by injecting the enzyme solution into an electrode cuvette and carried out at 25 °C.

One unit of piperonal-converting activity was defined as the amount of enzyme that catalyzed the formation of 1 μmol of piperonylic acid per min and the consumption of 1 μmol of O_2_ per min under the “standard assay A and B” conditions, respectively. Specific activity is expressed as units per milligram of protein. *k*_*cat*_ values were calculated using a *M*_r_ of 134,613 for PceSML. The protein concentrations were determined with a Nacalai Tesque protein assay kit using BSA as the standard, according to the method of Bradford^25^.

### Stoichiometry

The reaction mixture comprised 0.5 mM piperonal, 1 μg enzyme, and 100 mM KPB (pH 7.5), in a final volume of 200 μL. The reaction was started by the addition of piperonal and carried out at 25 °C. The reaction was stopped by the addition of an equal volume of 0.2 M HCl. Piperonal and piperonynlic acid were analyzed by HPLC according under the “standard assay A” conditions. H_2_O_2_ was analyzed by HPLC with a Shimadzu LC-10Avp system equipped with a Shodex SUGER KS-801 column (8.0 i.d. × 300 mm) under the following conditions: column temperature, 40 °C; isocratic elution; mobile-phase solvent (2 mM Na_2_SO_4_, 20 nM EDTA); flow rate, 0.75 ml/min; and electrochemical detection, ECD700S (Eicom, Kyoto, Japan). O_2_ was analyzed with an oxygen electrode with the same system as used for the measurement of O_2_ under the “standard assay B” conditions.

## Additional Information

**Accession codes:** The nucleotide sequence data for the 16 S rRNA gene and the piperonal-converting enzyme gene cluster reported in this paper appear in the DDBJ/GenBank database under accession numbers LC088725 and LC088726, respectively.

**How to cite this article**: Doi, S. *et al*. Discovery of piperonal-converting oxidase involved in the metabolism of a botanical aromatic aldehyde. *Sci. Rep.* **6**, 38021; doi: 10.1038/srep38021 (2016).

**Publisher's note:** Springer Nature remains neutral with regard to jurisdictional claims in published maps and institutional affiliations.

## Supplementary Material

Supplementary Information

## Figures and Tables

**Table 1:** Purification of the piperonal-converting enzyme.

Step	Total protein	Total activity	Specific activity	Yield	Fold
	*mg*	*units*	*units/mg*	*%*	
Cell free extract	174	371	2.13	100	1.1
(NH_4_)_2_SO_4_ (40–60%)	75.4	173	2.30	46.6	1.1
Butyl-Toyopearl 650 M	7.27	54.0	7.43	14.6	3.5
Ceramic Hydroxyapatite Type I	1.42	12.1	8.54	3.26	4.0
Resource Q	0.30	9.09	30.3	2.45	14
TSKgel Bioassist Q	0.003	0.164	54.7	0.044	26

**Table 2:** Electron acceptor for the piperonal-converting enzyme.

Electron acceptor	Relative activity
	*%*
O_2_	100
NAD^+^	N.D.
Ferredoxin	N.D.
Cytochrome *c*	N.D.
2,6-Dichlorophenol-indophenol	13
Benzoquinone	26

N.D., not detected.

**Table 3:** Substrate specificity.

Substrate	*K*_*m*_	*V*_*ma*x_	*k*_*cat*_/*K*_*m*_
	*mM*	*units/mg*	*s*^−1^*·mM*^−1^
Piperonal	0.019 ± 0.002	65.2 ± 2.2	7700
Formaldehyde	N.D.	N.D.	N.D.
Acetaldehyde	67.9 ± 14	97.3 ± 13	3
Propionaldehyde	0.37 ± 0.008	59.7 ± 4.1	360
Crotonaldehyde	0.26 ± 0.04	58.5 ± 3.2	510
Butyraldehyde	0.059 ± 0.01	68.7 ± 3.7	2600
Isobutyraldehyde	1.78 ± 0.45	79.0 ± 11	100
Valeraldehyde	0.05 ± 0.009	61.3 ± 2.6	2800
Isovaleraldehyde	0.049 ± 0.008	67.9 ± 3.9	3100
1-Hexanal	0.087 ± 0.01	67.7 ± 3.9	1700
Benzaldehyde	0.045 ± 0.009	65.1 ± 4.4	3200
Salicylaldehyde	0.024 ± 0.003	29.7 ± 1.4	2800
3-Hydroxybenzaldehyde	0.09 ± 0.01	59.6 ± 1.8	5600
4-Hydroxybenzaldehyde	0.024 ± 0.003	53.0 ± 2.7	1300
Cinnamaldehyde	0.024 ± 0.004	66.3 ± 3.7	6200
Protocatechualdehyde	0.31 ± 0.04	49.4 ± 2.1	360
Vanillin	0.097 ± 0.006	64.5 ± 1.1	1500
Xanthine	N.D.	N.D.	N.D.

The reaction was carried out as described under “*Methods*”. N.D., not detected.
