# Supplementary material for: Discovery of piperonal-converting oxidase involved in the metabolism of a botanical aromatic aldehyde
Source: Sci Rep. 2016 Dec 1;6:38021. doi: 10.1038/srep38021 (PMC5131310; doi:10.1038/srep38021)
Supplement: Supplementary Information [file srep38021-s1.pdf]

Supplementary information

Discovery of piperonal-converting oxidase involved in the metabolism of a botanical aromatic aldehyde

Shiori Doi¶, Yoshiteru Hashimoto¶, Chiaki Tomita, Takuto Kumano and Michihiko Kobayashi*

Institute of Applied Biochemistry, and Graduate School of Life and Environmental Sciences, The University of Tsukuba, 1-1-1 Tennodai, Tsukuba, Ibaraki 305-8572, Japan.

¶ These authors contributed equally to this work.

* To whom correspondence should be addressed.

Address correspondence to: Michihiko Kobayashi, Ph. D. (Professor)

Institute of Applied Biochemistry, and Graduate School of Life and Environmental Sciences, The University of Tsukuba, 1-1-1 Tennodai, Tsukuba, Ibaraki 305-8572, Japan.

Tel: +81-29-853-4628; Fax: +81-29-853-4605 (Institute); E-mail: kobayashi.m.fe@u.tsukuba.ac.jp

SI Methods

Materials

Piperonal was purchased from Nacalai Tesque Co., Inc. (Kyoto, Japan). Resource Q, Superose 12 HR10/30, HisTrap HP, HiTrap Desalting and an LMW calibration kit were obtained from GE Healthcare-Life Sciences (Buckinghamshire, UK). TOYOPEARL Butyl-650M and TSKgel BioAssistQ were purchased from Tosoh Co., Ltd. (Tokyo, Japan). Ceramic Hydroxyapatite Type I was obtained from Bio-Rad Laboratories (Hercules, CA, USA). Standard proteins for high-performance gel filtration chromatography were purchased from Oriental Yeast (Tokyo, Japan). All other biochemicals were standard commercial preparations.

Culture conditions for Burkholderia sp. CT39-3

Burkholderia sp. CT39-3 was inoculated into a subculture medium (pH 7.5) comprising 0.05% (w/v) KH_2PO_4, 0.05% (w/v) K_2HPO_4, 0.2% (w/v) NaCl, 0.05% (w/v) $MgSO_4{\cdot}7H_2O$, 0.001% (w/v) $FeSO_4{\cdot}7H_2O$, 0.5% (w/v) glycerol, 0.05% (w/v) malt extract, and 0.03% (w/v) piperonal (all final concentrations), and then incubated at 28°C for 96 h. The subculture was carried out at 28°C for 72 h with reciprocal shaking in a test tube containing 10 ml of the same medium. Then, 5 ml of the subculture was inoculated into a 2-l shaking flask containing 500 ml of the above-mentioned medium, followed by incubation at 28°C with reciprocal shaking. After 96 h incubation, the cells were harvested by centrifugation at 10,400 × *g* at 4°C and then washed twice with 10 mM KPB (pH 7.5).

The culture medium for investigating the function of piperonal oxidase was the screening medium containing 0.03, 0.07 or 0.10 % (w/v) piperonal, or glucose as a sole carbon source. *Burkholderia* sp. CT39-3 was inoculated (final conc., OD_{600} = 0.1), and cells were incubated at 28°C and 220 r.p.m.

Molecular mass determination

The purified enzyme sample was applied to a Superose 12 HR10/30 column (GE Healthcare UK Ltd.), attached to an ÄKTA purifier, and then eluted with 10 mM KPB containing 0.15 M KCl at the flow rate of 0.5 ml/min. The absorbance of the effluent was recorded at 280 nm. The molecular mass of the enzyme was calculated from the mobilities of the standard proteins, glutamate dehydrogenase (290 kDa), lactate dehydrogenase (142 kDa), enolase (67 kDa), adenylate kinase (32 kDa), and cytochrome *c* (12.4 kDa).

Plasmids, strains, and medium

Escherichia coli SCS110 (Agilent Technologies) was used as the host for pBBR1MCS2 (1) plasmids. *E. coli* transformants were grown in 2× YT medium containing 50 μg/ml kanamycin (2), unless otherwise noted.

DNA manipulations

Restriction endonucleases, DNA polymerase, and T4 DNA ligase were purchased from Toyobo Co., Ltd. (Osaka, Japan). Nucleotides were sequenced by the dideoxy-chain terminating method using an Applied Biosystems 3730×1 DNA analyzer (Applied Biosystems, Foster City, CA, USA). Unless otherwise stated, DNA manipulations were performed essentially as described by Maniatis et al. (2).

Preparation of Burkholderia sp. CT39-3 competent cells for electroporation

The subculture was carried out at 28°C with reciprocal shaking in a test tube containing 5 ml of the 2× YT medium until the OD_{600} reached ~ 0.5. Then, the subculture was diluted to an OD_{600} of 0.01 in 50 ml of SOB medium (2) containing 10 mM $MgSO_4$, 10 mM $MgCl_2$, and 0.3% (w/v) glycine, followed by incubation at 28°C with reciprocal shaking until the OD_{600} reached 0.05. After incubation, the cells

were harvested by centrifugation at 8,000 × *g* at 4°C. The pellet from a 100 ml culture was resuspended in 20 ml ice-cold FSB (pH 7.5) (2), incubated in an ice bath for 10 min, and then spun down three times. Then, the cells were washed twice with ice-cold 0.5 M sucrose and the suspension was centrifuged. After washing with 0.5 M sucrose, the cells were resuspended in 200 μl of ice-cold 0.5 M sucrose containing 10% (w/v) glycerol. Aliquots (100 μl) were stored at – 80°C until use (3-4).

Electroporation

Cells were thawed in ice, and transferred to a sterile, pre-chilled cuvette (interelectrode distance of 0.2 cm). Plasmid DNA (15 μg) and Ocr (Type One Restriction Inhibitor; Epicentre, Madison, WI, USA) were added to the cuvette, followed by mixing by inversion. Then, the cuvette was placed in a Gene Pulser Xcell (Bio-Rad Labolatories), and the cells were subjected to a pulse of 2.5 kV and 50 μF. Immediately after pulse application, the cell suspension was diluted with 1 ml of SOC medium (2). The cells were then transferred to a tube and incubated at 28°C for at least 4 h without any antibiotic. After incubation, aliquots were spread-plated on 2× YT medium containing 50 μg/ml kanamycin.

Analytical methods

Protein concentrations were determined with a Nacalai Tesque Co., Inc. protein assay kit, with bovine serum albumin as the standard. SDS-PAGE was performed in a 12% polyacrylamide slab gel according to Laemmli (5). The gel was stained with Coomassie brilliant blue R-250. The relative molecular mass of the enzyme was calculated from the mobilities of the marker proteins, phosphorylase *b* (94 kDa), bovine serum albumin (67 kDa), ovalbumin (43 kDa), carbonic anhydrase (30 kDa), soybean trypsin inhibitor (20.1 kDa), and α-lactalbumin (14.4 kDa).

UV-Vis spectra were obtained with a Shimadzu UV-1700 spectrophotometer (Kyoto, Japan) at room temperature. Enzymes were dialyzed against 100 mM KPB (pH 7.5) and then prepared at 2.2

mg/ml.

The N-terminal amino acid sequences were determined with samples electroblotted onto a polyvinylidene difluoride (PVDF) membrane after SDS-PAGE using a Procise protein sequencer (Applied Biosystems).

Expression and purification of the recombinant piperonal-converting enzyme

DNA fragments containing the coding regions (*pceSML*) of the enzyme (*piperonal-converting enzyme)* were amplified by PCR with genomic DNA extracted from the CT39-3 strain as a template. The following two oligonucleotide primers were used: sense primer, 5'-CATATCGAAGGTCGTCATATGCCACAACGTCACATCCCCCAATCG-3' containing an *Nde*I recognition site (underlined); and antisense primer, 5'-GCTCTAGAACTAGTGGGATCCTTACAGCAAGTCCTCGATCATCACCGGC-3' containing a *Bam*HI recognition site (underlined). Also, DNA fragments containing the His•tag coding sequence were amplified by PCR with pET-16b (Novagen) as a template. The following two oligonucleotide primers were used: sense primer, 5'-AAGGGAACAAAAGCTGGTACCGGGGAATTGTGAGCGGATAACAATTCCCC-3' containing a *Kpn*I recognition site (underlined); and antisense primer, 5'-CATATGACGACCTTCGATATGGCCGCTGC-3' containing an *Nde*I recognition site (underlined). Both amplified DNA fragments were inserted into the *Kpn*I and *Bam*HI sites of pBBR1MCS2 using an In-Fusion® HD Cloning Kit (Clontech® Laboratories, Inc., Mountainview, CA, USA), and checked by DNA sequencing. The resulting plasmid, pBBR-His-*pceSML,* expresses piperonal oxidase as an N-terminal His•tag fusion with PceS. In this construct, the *pceSML genes* were under the control of the *lac* promoter.

Burkholderia sp. CT39-3 was transformed with pBBR-His-*pceSML*, and the recombinant cells

were used for the overproduction and purification of a recombinant piperonal-converting enzyme. The transformed cells were incubated with reciprocal shaking at 28°C in 100 ml of 2× YT medium containing 50 μg/ml kanamycin. After overnight cultivation, 10 ml of the culture was inoculated into 1 liter of the same medium. IPTG was then added to a final concentration of 1 mM to induce the *lac* promoter, followed by incubation with shaking at 28°C for 72 h.

All purification procedures were performed at 0-4°C. KPB (pH 7.5) was used throughout the purification. Centrifugation was carried out for 30 min at 13,000 × *g*.

Step 1. Preparation of a Cell-free Extract. Washed cells from 0.5 liters of a culture were resuspended in 50 ml of 100 mM buffer and then disrupted by sonication at 190 W for 30 min with an Insonator model 201M. The cell debris was removed by centrifugation.

Step 2. HisTrap HP Column Chromatography. The resulting supernatant solution was applied to a HisTrap HP column (1 ml; GE Healthcare UK Ltd.), attached to an ÄKTA purifier (GE Healthcare UK Ltd.) and equilibrated with 100 mM buffer. Protein was eluted from the column with 20 ml of the same buffer, the concentration of imidazole being increased linearly from 0 to 1 M. The active fractions were pooled and the homogeneity of the purified protein was confirmed by SDS-PAGE.

Genome sequencing of strain CT39-3

Genomic DNA was prepared from *Burkholderia* sp. strain CT39-3 as follows: the strain was cultured at 28°C for 24 h in 500 ml of 2× YT medium with reciprocal shaking. Cells were harvested by centrifugation, washed with 10 mM Tris buffer (pH 8.0) containing 1 mM EDTA and 100 mM NaCl, and then suspended in 15 ml of the same buffer. The suspension was incubated with 7 mg/ml of lysozyme at 37°C for 3 h, and then 1 ml of 0.25 mM EDTA (pH 8.0), 0.5 ml of 10% (w/v) SDS and 4 mg of proteinase K (Wako Pure Chemical Industries, Ltd.) were added to the solution, followed by incubation at room temperature for 3 h. 5.3 ml of 5 M NaCl was added to the solution. DNA was purified by

extraction of the lysate with phenol/chloroform/isoamylalcohol (25/24/1; v/v/v), and then precipitation with isopropanol. The solution was subjected to equilibrium centrifugation in a CsCl-ethidium bromide gradient, and the fraction containing genomic DNA was pooled, extracted with *n*-butanol to remove ethidium bromide, and then precipitated with ethanol. The draft genome sequencing of strain CT39-3 was performed using an Illumina Hiseq platform (Hokkaido System Science Co., Ltd., Sapporo, Japan). The annotation of the contigs was performed with the Microbial Genome Annotation Pipeline (http://www.migap.org).

Identification of the enzyme flavin cofactor

For HPLC-based identification of the flavin cofactor of the enzyme, the enzyme was precipitated by the addition of 5% perchloric acid, sedimented at 10,000 × *g*, and then analyzed using a Shimadzu LC-10A HPLC system (Kyoto, Japan) with a Cosmosil 5C_{18}-AR-II column (reversed-phase, 4.6 × 150 mm; Nacalai Tesque Co., Inc.) at 35°C. Isocratic separation was performed at a flow rate of 1.0 ml/min using methanol/0.2% KH_2PO_4 (20:80, all by volume) as the mobile phase. The absorbance was measured at 260 nm for flavins. Flavin-adenine-dinucleotide (FAD) and flavin-mononucleotide (FMN) were used as standards.

Analysis of cytidine monophosphate in MCD cofactor

Cytidine monophosphate (CMP) was released from MCD by 15 min incubation after the addition of 5% (v/v) sulfuric acid. The reaction product was sedimented at 10,000 × *g* and then separated by HPLC using a TSKgel Amide80 column (reversed-phase, 4.6 × 150 mm; Nacalai Tesque Co., Inc.) equilibrated in 30% 10 mM ammonium acetate and 70% acetonitrile at an isocratic flow rate of 1 ml/min at 35°C. CMP was quantified relative to standard CMP solutions (6).

Metal analysis

All glassware was exhaustively rinsed with distilled water before use. Prior to analysis, the enzyme was dialyzed against 10 mM KPB (pH 7.5). The enzyme sample containing 1.1 mg PceSLM/ml was analyzed with an inductivity coupled radiofrequency plasma spectrometer, Shimadzu ICPS-8100 (27.120 MHz; Kyoto, Japan). The metal contents of the enzyme sample were determined from the calibration curves for standard solutions.

Detection of $O_2^{\bullet -}$ formation using the cytochrome c assay

Formation of superoxide ($O_2^{\bullet -}$) was detected as the reduction of ferricytochrome *c* at 550 nm at room temperature. The total volume of the reaction mixture was 1 ml, which contained 0.1 mM EDTA, 0.03 units enzyme and 0.5 mM piperonal as a substrate. Piperonal was added to initiate the reaction and the absorbance change at 550 nm was recorded using a Shimadzu UV-1700 spectrophotometer (Kyoto, Japan). Similar experiments were performed with xanthine oxidase (XOD) (Oriental Yeast Co., Ltd, Tokyo, Japan) under identical conditions except that 0.03 units xanthine was used as a substrate instead of piperonal (7-8).

Substrate specificity

The following compounds were examined as to substrate specificity: piperonal (0.00625~2 mM), formaldehyde (0.123~12.3 mM), acetaldehyde (2~40 mM), propionaldehyde (0.2~20 mM), crotonaldehyde (0.05~20 mM), butyraldehyde (0.025~2 mM), isobutyraldehyde (0.2~20 mM), valeraldehyde (0.025~2 mM), isovaleraldehyde (0.025~2 mM), 1-hexanal (0.05~20 mM), benzaldehyde (0.02~0.2 mM), salicylaldehyde (0.025~40 mM), 3-hydroxybenzaldehyde (0.01~2 mM), 4-hydroxybenzaldehyde (0.05~ 2 mM), cinnamaldehyde (0.0125~2 mM), protocatechualdehyde (0.05~2 mM), vanillin (0.00625~2 mM), and xanthine (2 mM). The level of O_2 consumption during the

enzymatic reaction was determined with an oxygen electrode under the "standard assay B" conditions given in "*Enzyme assays*".

Determination of minimum inhibitory concentration (MIC)

At first, growth inhibition by piperonal or ethanol (which is used as the solvent for piperonal) was investigated in broth microdilution assays (9), using a 5 ml inoculum of each lineage, in a concentration of about 10^5 CFU/ml, in 2YT medium. Next, the minimum inhibitory concentration (MIC) of piperonal was determined using microdilution plates (containing various amounts of piperonal) with 9 wells. In each well, 2 μl of each sample solution was added on the plate. The final piperonal concentrations varied 6.25 and 800 μg/ml. The plates were incubated at 28°C for 24 h or at 37°C for 4 days. The MIC was defined as the lowest concentrations necessary for the inhibition growth.

SI Results

Optimum culture conditions for formation of the piperonal-converting enzyme

To increase the enzyme activity in the cells, we investigated several sets of culture conditions for strain CT39-3. A subculture of strain CT39-3 was carried out at 28°C for 72 h with reciprocal shaking in a test tube containing 10 ml of the screening medium. Next, 0.1 ml of the cell suspension was inoculated into a test tube containing 10 ml of one of the various media to be examined in each experiment. Each cultivation was carried out at 28°C for 72 h with reciprocal shaking.

First, the effects of various carbon sources were examined. Of different carbon sources, 0.2% (w/v) was added to the medium in the absence or presence of 0.03, 0.05, 0.1, or 0.15% (w/v) piperonal as follows: glucose, maltose, mannitol, glycerol, sucrose, lactose, and galactose. Among the tested culture media, the highest specific activity was observed in the medium containing 0.03% (w/v) piperonal in the presence of glycerol. Second, the effects of various nitrogen sources were examined. The following compounds were tested as nitrogen sources: casamino acid, malt extract, meat extract, tryptone, beef extract, NH_4Cl, NH_4HPO_4, NZ amine, peptone, yeast extract, bonito extract, $(NH_4)_2SO_4$, and urea. Each of the various compounds was added, to the final concentration of 0.2% (w/v), to the medium instead of $(NH_4)_2SO_4$. The replacement of $(NH_4)_2SO_4$ with malt extract increased the enzyme activity 1.5-fold compared with that in the medium with $(NH_4)_2SO_4$. Thus, malt extract was selected as the nitrogen source for the following experiments. Next, various concentrations of glycerol and malt extract were added to the medium. The best concentrations were 0.5% (w/v) for glycerol and 0.05% (w/v) for malt extract, respectively, being 1.8-fold compared with that in the initial medium. Based on the above results, a medium containing 0.03% (w/v) piperonal, 0.5% (w/v) glycerol, 0.05% (w/v) malt extract, 0.05% (w/v) KH_2PO_4, 0.05% (w/v) K_2HPO_4, 0.2% (w/v) NaCl, 0.05% (w/v) $MgSO_4 \cdot 7H_2O$, and 0.001% (w/v) $FeSO_4 \cdot 7H_2O$ (pH 7.5) was found to be the most suitable medium for the preparation of cells exhibiting high piperonal-converting activity.

To increase the piperonal-converting ability, various culture conditions were examined (Table S1). Strain CT39-3 was inoculated into a 2-L shaking flask containing various volumes of the above-mentioned medium, followed by incubation at 28°C. As for culture volume conditions, higher specific activities were found in cells grown in the 0.5 L of the medium. Specific activity gradually increased until 96 h. Although a further significant increase in the enzyme activity was not obtained during incubation for longer than 96 h, the maximum activity was observed at 144 h cultivation. To obtain a large amount of cells, repeated large-scale cultures using a 2-L shaking flask was required. Therefore, 96 h incubation in a flask containing 0.5 L culture medium were taken as the optimum conditions. Under these conditions, the level of piperonal-converting activity in a cell-free extract was 4.39 units/mg; i.e., the piperonal-converting activity was increased 4.8-fold compared with that under the initial conditions.

Characterization of piperonal as an antimicrobial compound

To investigate the antimicrobial activity of piperonal, various Gram-positive and Gram-negative bacteria were grown in the presence of piperonal or ethanol. Growth of some bacteria was inhibited by piperonal, and we determined the minimum inhibitory concentration (MIC) of piperonal against them. As a result, piperonal showed the antimicrobial activity against at least the following 4 bacteria, *Exiguobacterium acetylicum* NBRC12246^T, *Mycobacterium diernhoferi* NBRC14797^T, *Nocardia farcinica* NBRC3423^T, and *Rhodobacter sphaeroides* NBRC12203^T. (MIC: 50, 100, 800, and 800 μg/ml).

SI Discussion

Physiological role of the piperonal-converting enzyme

In order to determine the physiological role of the piperonal-converting enzyme, we cultivated *Burkholderia* sp. CT39-3 in the medium containing glucose or piperonal as the sole carbon source. CT39-3 grew well as the concentration of glucose in the medium became higher. On the contrary, CT39-3 grew slowly in the medium containing a low amount of piperonal [0.03% (w/v)]. The growth of CT39-3 was increasingly inhibited as the concentration of piperonal in the medium became higher (Fig. 5A). Interestingly, the piperonal-converting enzyme activity was observed under all conditions tested, even in the presence of glucose [0.026, 0.227 and 0.044 units/mg for 0.10, 0.07 and 0.03% (w/v) piperonal, respectively; and 0.043, 0.077 and 0.119 units/mg for 0.10, 0.07 and 0.03% (w/v) glucose, respectively]. Moreover, the concentration of piperonal did not affect the activity, namely the formation of the piperonal-converting enzyme. These findings suggest that the enzyme is not inducible but is expressed constitutively. That the bacterial growth depended on the concentration of piperonal suggests that a high level [0.1% (w/v)] of piperonal is toxic for strain CT39-3. A lower amount of piperonal contributed as a carbon source and restored growth of strain CT39-3. Moreover, analysis of the substrate specificity of the piperonal-converting enzyme showed broad substrate specificity with various aldehydes, whereas purines were not oxidized by the piperonal-converting enzyme. These findings indicate that the piperonal-converting enzyme is not a xanthine oxidase involved in purine catabolism, and that the physiological role of the piperonal-converting enzyme is the utilization of various aldehyde compounds and/or their detoxification into less toxic acids.

Comparison of the piperonal-converting enzyme with other aldehyde oxidoreductases

In *E. coli,* the gene cluster for the periplasmic aldehyde oxidoreductase consists of *paoABCD*. PaoA binding two distinct [2Fe-2S] clusters, PaoB binding FAD, and PaoC containing molybdopterin

cytosine dinucleotide cofactor (MCD) are subunits of the trimeric aldehyde oxidoreductase, respectively. The product of *paoD*, which is located just downstream of the structural genes, is predicted to be involved in formation of MCD and its insertion into PaoC, because expression of *paoABC* without *paoD* results in the formation of apo-PaoABC, in which MCD is not included (10). The gene cluster for another aldehyde oxidoreductase derived from *Methylobacillus* sp. KY4400 consists of *orf12345*(6). The products of ORF1, ORF2 and ORF3 contain the [2Fe-2S] clusters, FAD and MCD, respectively. The deduced amino acid sequence of ORF5 located downstream of the structural genes shows sequence similarity to PaoD, and might be involved in maturation of MCD and its insertion into the product of ORF3. The deduced amino acid sequence of ORF4 exhibits weak sequence similarity only to some unknown proteins, thus, the function of *orf4* is unclear (11). As for the piperonal-converting enzyme (PceSML), PceS, which shows sequence similarity to the subunit containing the [2Fe-2S] clusters for aldehyde oxidoreductases, exhibits the characteristic absorption spectrum (peaks at 311, 319, 420, 470, and 550 nm) of the [2Fe-2S] centers. PceM shows sequence similarity to the subunit containing FAD, and had an absorption spectrum exhibiting a cofactor absorption peaks derived from the FAD absorption maxima at 360 nm and 450 nm. PceL shows sequence similarity to the subunit containing molybdopterin. Considering the above all together, the piperonal-converting enzyme is suggested to contain the [2Fe-2S] clusters, FAD and molybdopterin.

SI References

1. Kovach, M. E. *et al* Four new derivatives of the broad-host-range cloning vector pBBR1MCS, carrying different antibiotic-resistance cassettes. *Gene* **166**, 175-176, doi:10.1016/0378-1119(95)00584-1 (1995).

2. Sambrook, J., Fritsch, E. F. & Maniatis, T. *Molecular Cloning: A Laboratory Manual*, 2nd Ed., (Cold Spring Harbor Laboratory, Cold Spring Harbor, NY, 1989).

3. Hayashi, M., Maeda, Y., Hashimoto, Y. & Murooka, Y. Efficient transformation of *Mesorhizobium huakuii* subsp. *rengei* and *Rhizobium* species. *J. Biosci. Bioeng.* **89**, 550-553, doi:10.1016/S1389-1723(00)80055-9 (2000).

4. Dubarry, N., Du, W., Lane, D. & Pasta, F. Improved electrotransformation and decreased antibiotic resistance of the cystic fibrosis pathogen *Burkholderia cenocepacia* strain J2315. *Appl. Environ. Microbiol.* **76**, 1095-1102, doi:10.1128/AEM.02123-09 (2010).

5. Laemmli, U. K. Cleavage of structural proteins during the assembly of the head of bacteriophage T4. *Nature* **227**, 680-685, doi:10.1038/227680a0 (1970).

6. Neumann, M. *et al* A periplasmic aldehyde oxidoreductase represents the first molybdopterin cytosine dinucleotide cofactor containing molybdo-flavoenzyme from *Escherichia coli*. *FEBS J.* **276**, 2762-2774, doi:10.1111/j.1742-4658.2009.07000.x (2009).

7. Kundu, K. K., Hille, R., Velayutham, M. & Zweier, L. J. Characterization of superoxide production from aldehyde oxidase: an important source of oxidants in biological tissues. *Arch. Biochem. Biophys*. **460**, 113-121, doi:10.1016/jab.2006.12.032 (2007).

8. Kundu, K. K., Hille, R., Velayutham, M. & Zweier, L. J. Aldehyde oxidase functions as a superoxide generating NADH oxidase: an important redox regulated pathway of cellular oxygen radical formation. *Biochemistry*. **51**, 2930-2939, doi:10.1021/bi3000879 (2012).

9. Lima, V.N. *et al* Antimicrobial and enhancement of the antibiotic activity by phenolic compounds: Gallic acid, caffeic acid and pyrogallol. *Microb. Pathog.* **99**, 56-61, doi:10.1016/j.micpath.2016.08.004. (2016).

10. Neumann, M. & Leimkuhler, S. The role on system-specific molecular chaperones in the maturation of molybdoenzymes in bacteria. *Biochem. Res. Int.* 850924, doi:10.1155/2011/850924 (2011).

11. Yasuhara, A., Akiba-Goto, M. & Aisaka, K. Cloning and sequencing of the aldehyde oxidase gene from *Methylobacillus* sp. KY4440. *Biosci. Biotechnol. Biochem.* **69**, 2435-2438, doi:10.1271/bbb.69.2435 (2005).

Table S1. *Piperonal-converting enzyme activity of Burkholderia sp. CT39-3 under various culture conditions.*

Culture volume (*L*)	Specific activity (*units/mg*)													
	Cultivation time (*h*)													
	12	24	36	48	60	72	84	96	108	102	132	144	156	168
0.1	N.D.	0.15	0.64	2.05	2.30	2.77	2.63	3.18	3.17	3.97	3.02	3.07	3.47	4.16
0.5	N.D.	N.D.	0.49	1.76	2.67	3.06	3.70	4.39	4.46	4.50	4.91	5.02	4.94	4.63
1.0	N.D.	N.D.	0.30	1.22	2.08	2.52	2.61	3.07	3.35	3.06	3.57	3.74	4.03	3.85

N.D., not detected.
